# Geneotype and phenotype in 20 patients with glycogen storage disease type Ia

**DOI:** 10.1186/1687-9856-2013-S1-P175

**Published:** 2013-10-03

**Authors:** Cuili Liang, Li Liu, Huiying Sheng, Minyan Jiang, Xi Yin

**Affiliations:** 1 Department of Pediatric Endocrinology and Metabolism, Guangzhou Women and Children’ Medical Center. Guangzhou, Guangdong, China

## Background

Glycogen Storage Disease Type Ia (GSD Ia) is a group of autosomal recessive inborn errors of metabolism that is caused by deficiency in glucose-6-phosphatase,and it is the major subtype of Liver Glycogen Storage Disease(LGSD) cases. Patients afflicted with GSD Ia cannot maintain glucose homeostasis and manifest hypoglycemia, hepatomegaly, lactic academia. However, we cannot separate GSD Ia form LGSD through clinical manifestations and routine laboratory tests, except for analysis of G6PC gene mutation and assay of glucose-6-phosphatase enzyme activity, which is an invasive method. Therefore, the analysis of G6PC gene mutation is an important method for diagnosing GSD Ia.

## Objective

To investigate the G6PC gene mutations in patients with Glycogen Storage Disease type Ia (GSD Ia),and analyze the relationship of its genotype and phenotype.

## Methods

We diagnose 48 patients with LGSD by clinical manifestations, laboratory tests and glucagon test. The entire coding region of the G6PC gene from peripheral blood was screened by PCR combined with direct DNA sequencing. The G6PC genes of 50 unrelated healthy children were investigated as comparisons to rule out gene polymorphism. We used software named DNAMAN to analyze their conservatism through many species homology comparison.

## Results

Among 48 patients, there were 41.67 %(20/48) of them were found to have G6PC gene mutations. Totally eight types of G6PC gene mutations were detected, which were 727G>T, R83H, H119L, L173P, I341N, V88Xfs254, C109T and W87Xfs270,and 727G>T and R83H were more frequent, with frequencies of 37.50%(15/40) and 22.50%(9/40), respectively. C109T and W87Xfs270 were never reported before. Our clinical and routine laboratory data showed that the patients manifested the same phenotype even with different genotype.

## Conclusions

Our study revealed 727G>T and R83H mutations were prevalence in Chinese patients with GSD Ia. C109T and W87Xfs270 might be novel pathogenic mutations. There was no clear relationship between genotype and phenotype.

